# Involving Patients and Families in the Analysis of Suicides, Suicide Attempts, and Other Sentinel Events in Mental Healthcare: A Qualitative Study in The Netherlands

**DOI:** 10.3390/ijerph15061104

**Published:** 2018-05-29

**Authors:** Renée Bouwman, Bert de Graaff, Derek de Beurs, Hester van de Bovenkamp, Ian Leistikow, Roland Friele

**Affiliations:** 1 NIVEL, P.O. Box 1568, 3500 BN Utrecht, The Netherlands; d.debeurs@nivel.nl (D.d.B.); r.friele@nivel.nl (R.F.); 2 Erasmus School of Health Policy & Management, Erasmus University Rotterdam, P.O. box 1738, 3000 DR Rotterdam, The Netherlands; degraaff@eshpm.eur.nl (B.d.G.); vandebovenkamp@eshpm.eur.nl (H.v.d.B.); ip.leistikow@igj.nl (I.L.); 3 Dutch Healthcare and Youth Inspectorate, 3521 AZ Utrecht, The Netherlands; 4 TRANZO (Scientific Centre for Care and Welfare), Faculty of Social and Behavioural Sciences, Tilburg University, 5037 DB Tilburg, The Netherlands

**Keywords:** mental healthcare, suicide, suicide attempt, participation, sentinel event, incident, family, bereaved, incident management, incident analysis, root cause analysis

## Abstract

Involving patients and families in mental healthcare is becoming more commonplace, but little is known about how they are involved in the aftermath of serious adverse events related to quality of care (sentinel events, including suicides). This study explores the role patients and families have in formal processes after sentinel events in Dutch mental healthcare. We analyzed the existing policies of 15 healthcare organizations and spoke with 35 stakeholders including patients, families, their counselors, the national regulator, and professionals. Respondents argue that involving patients and families is valuable to help deal with the event emotionally, provide additional information, and prevent escalation. Results indicate that involving patients and families is only described in sentinel event policies to a limited extent. In practice, involvement consists mostly of providing aftercare and sharing information about the event by providers. Complexities such as privacy concerns and involuntary admissions are said to hinder involvement. Respondents also emphasize that involvement should not be obligatory and stress the need for patients and families to be involved throughout the process of treatment. There is no one-size-fits-all strategy for involving patients and families after sentinel events. The first step seems to be early involvement during treatment process itself.

## 1. Introduction

The analyses of sentinel events such as suicide and suicide attempts are key moments for drawing lessons for quality improvement in healthcare [1,2,3,4]. Sentinel events are defined as incidents that are wholly or partially caused by poor quality of care. From this perspective, analyses of the pathway before sentinel events could provide lessons for improvement [4]. The potential benefits of involving patients and families or friends (from here summarized as “family”) in the analysis of sentinel events are recognized by experts [5].

Within hospital care, there have been some studies on involving patients (and family) in analyses of sentinel events [6]. Studies show that the patient and/or family can provide information about the sentinel event that may help organizations learn [1,2,6]. Moreover, being involved can help patients and families in their grieving process as they learn about the context of the event and perceive that something is being done to prevent it from happening again. It is argued that involvement of patients and family might help to reduce guilt, frustration, and anger for healthcare professionals themselves. Reported benefits for the healthcare organization include increasing its legitimacy and demonstrating the organization’s commitment to learn and improve. Importantly, it can help the organization restore the relationship of trust with the patient [2]. Disadvantages have also been reported of involving patients and family in analyses of sentinel events in hospital care. For instance, if a sentinel event has resulted in death or significant harm to the patient, involving patients or family can lead to more damage by reliving the trauma instead of helping them through the grieving process. Participation in the event analysis can thus make the normal grieving process worse. There may also be disadvantages for staff. For them it can be an uncomfortable, emotional experience if patients are involved in analyzing the event, and it can have a negative influence on dialog between staff members during the analysis, for example because they are afraid to be open with the patient or family. Professionals can become defensive or too cautious because of fear of recurrence or legal consequences [2].

Involving patients and family during treatment of patients in mental healthcare is becoming more commonplace [7,8,9]. Research has been conducted on the involvement of patients and family during treatment. Within mental healthcare, joint decision-making is found to be essential to come to the most appropriate and most successful treatment [10]. The professional carer, patient, and family each bring their own expertise, and decide together how the problem will be tackled [10]. In addition, the chances of success of the treatment are found to increase when family are involved [11,12,13]. Family members know things about the patient that may be important for treatment and risk assessment, and often act as the first safety net for the patient [7,14,15,16]. In addition, family may provide informal care and can take on the role of case manager [7]. Patients and families are thus increasingly playing an active role in treatment decisions. In addition they are increasingly involved at the level of organizational policy making in mental healthcare [7,8,15]. The idea is that this can improve the quality of care provided.

However, when sentinel events do occur in mental healthcare, the way in which patients and families are involved in the analysis of the event is unclear. Currently, many questions remain about reasons why and how best to involve them. The main question of this study was therefore how patients and families are involved in sentinel event analyses in practice and what the main bottlenecks and contributory factors are.

To the best of our knowledge, no earlier studies have examined this issue in the field of mental healthcare. To explore the involvement of patients and families after the occurrence of a sentinel event in mental healthcare we examined whether and how patients and families are involved in the analysis of, and formal reporting on a sentinel event including suicide (attempts). We did so by conducting a qualitative study on this issue in the Netherlands. The Netherlands is an excellent case for studying this issue as recent laws and guidelines explicitly emphasize the value of the participation of patients and families in maintaining the quality of care [17,18,19]. This includes involvement in sentinel event analyses. The new Dutch guideline on the assessment and treatment of suicidal behavior argues that the involvement of and cooperation with family is as important as informing them about the patient being suicidal and the safety and continuity of care [20]. More recently, a generic guideline on how to involve family during treatment within both general practice and specialized mental healthcare settings was released, stressing the need for the involvement of family in every phase of the treatment [10].

When an incident occurs, Dutch law states that care organizations have to evaluate what happened and whether the quality of care (or shortcomings in it) caused the incident, including suicide and suicide attempts. If a sentinel event resulted in death or other serious outcome for a patient and the conclusion is that this outcome was related to quality of care, the mental healthcare organization has to report this to the Dutch Health & Youth Care Inspectorate (see Box 1 about this procedure). In this study, we included all types of sentinel events, including suicide and suicide attempts.

Box 1 **Information about reporting sentinel events to the national regulator in the Netherlands.**

According to the Dutch Quality, Complaints and Disputes in Healthcare Act (Wkkgz), healthcare providers must report ‘sentinel events’ (in Dutch: *calamiteiten*) to the national regulator (Health and Youth Care Inspectorate). The law describes a sentinel event as “an unintended or unexpected event, which relates to the quality of the care and which led to the death of or a serious harmful consequence for a patient” [4]. The European Commission uses a similar definition [21].

Care providers have to determine whether an adverse event meets the criteria of the legal definition. If not, it is classified as an incident that does not need to be reported and can be internally evaluated by care providers.

Sentinel events must be reported to the regulator within three working days. If it is not yet clear if a situation is a sentinel event, the care provider has 6 weeks to investigate further. If during this investigation it is concluded that an event was a sentinel event, this must be reported to the Inspectorate within three working days. The care provider must then carry out an analysis into the sentinel event themselves, unless the inspectorate decides to investigate.

In 2014, reported sentinel events in mental healthcare included suicides (the largest group of reported events at 41%), problems related to somatic care, medication errors, poor professional performance, aggression, shooting and stabbing incidents, and sexual intimidation/abuse. Suicides and suicide attempts with serious injuries must be reported when the patient was involuntary admitted (A person who is a danger to himself or those around him can be admitted involuntarily (committed) to a mental health institution. The procedure for admitting a person involuntarily is laid down in the Psychiatric Hospitals (Committals) Act (BOPZ).) to mental healthcare [22].

## 2. Materials and Methods

**Figure 1:**
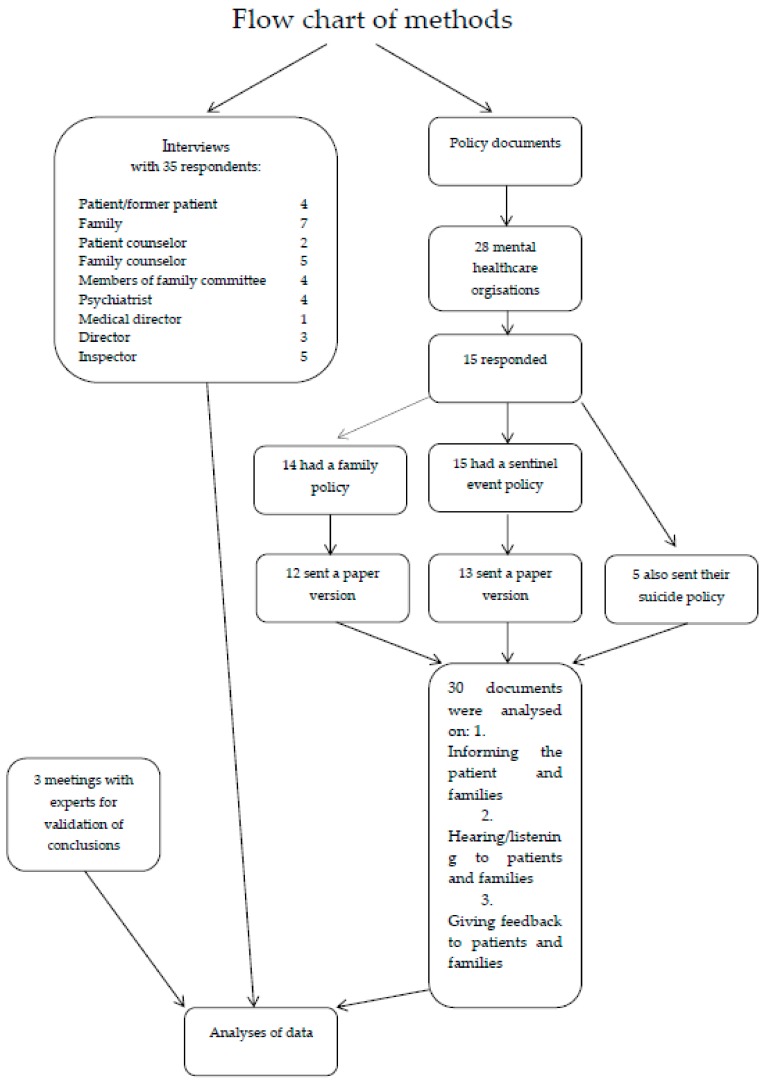
Flow chart of methods used in this study.

We conducted an explorative multi-method qualitative study consisting of document analysis and semi-structured interviews with various stakeholders (see Flow chart for specification) involved with sentinel events (see Figure 1 for Flow chart). Our study was carried out in the context of an ongoing research partnership with the Dutch Health & Youth Care Inspectorate. This research was carried out within the Academic Collaborative Centre on Supervision, where researchers from four research institutes cooperate with the Inspectorate. We included all types of sentinel events in order to gather rich and deep understanding of the experiences of involved persons relating to various events.

### 2.1. Policy Documents

The first step of the research was the analysis of policy documents from Dutch mental healthcare organizations and national policy documents. From these, we made an overview of the key aspects of involving patients and families in the evaluation of sentinel events, such as what procedures exist, which timeframes apply, what criteria exist for the analyses of sentinel events, which persons are involved etc., as currently laid down in guidelines, protocols, and other policies. This let us get a clearer picture of the extent to which the subject is already embedded in care organizations.

#### 2.1.1. Data Collection

We contacted a convenience sample of 28 mental healthcare institutions (which are further divided into smaller locations) from all parts of the Netherlands. 24 were among the largest mental health care institutions in the country.

In 2014, 70% of the turnover in mental healthcare came from 34 large mental healthcare institutions [23]. We approached them by letter asking them to send their family and sentinel events policies and to answer four questions about them (if the policy document is available, when it was drawn up, when it was reviewed for the last time and how frequently it is reviewed). A family policy is defined as a document that describes in what way family of patients will be approached and involved by the institution, with the aim of improving the care process. A sentinel event policy is defined as a plan on what to do when a sentinel event occurs. The latter definition also applies in the case of suicide and suicide attempts.

Of the 28 mental healthcare organizations approached, 15 responded to our request to send the protocols on family and sentinel event policies. All responding organizations indicated that they had a sentinel event policy (sent by 13) and 14 institutions indicated that they had a family policy (sent by 12). The sentinel event policies were drawn up between 2010 and 2017 (median 2014). Seven organizations indicated that they regularly evaluate the sentinel event policy. The family policies were drawn up between 2006 and 2016 (median 2013) and four organizations indicated that they regularly reviewed this. Five organizations also communicated their suicide policy.

#### 2.1.2. Analyses of Policy Documents

A content analysis was performed on the policy documents to assess the degree of the involvement of patients and family members [24]. Two researchers separately scored the occurrence of three categories of involving patients and family in the sentinel event and family policies by means of a score list (see Table 1). This distinction was made, based on previous research into patient involvement in regulation of quality of care. That study distinguished the three categories after an analysis of literature, policy documents, interviews, and focus groups with national and international inspectors, and patients [9]. That study focused on participation in healthcare quality regulation more generally, so more specific ways of involvement in sentinel analysis are not specified in the three categories:

1. **Informing** the patient and family members (about what happened, and what is going to happen further)

2. **Hearing/listening** to patients and relatives about their perspective on the event (hearing their perspective on what has happened and including this information in the sentinel event analysis)

3. **Giving feedback** to patients and families (feedback of results of event analysis and possibility of feedback on the report).

The scores were then compared and discussed, after which an agreement was reached. A number of additional characteristics were scored (Table 1).

### 2.2. Interviews

We conducted interviews with different groups of stakeholders involved with sentinel events in order to maximize the breadth of experiences with involving (or not) of patients and family. Based on a preliminary study of the literature, we approached stakeholders through professional organizations, interest groups, and patient organizations using a snowball method. Respondents we spoke to referred us to other relevant stakeholders to include in our study.

In total 35 people were interviewed including patients, family, family counselors, directors and inspectors (Flow chart). The goal of these interviews was to gain a picture of the experiences with the involvement of patients and families in cases of the analysis of sentinel events and their ideas about if and how to encourage this involvement. The interviews were semi structured, and a topic list was used as a guide line for the interviewer. Topics included the role of patients and families in sentinel events, what the considerations are in whether or not to involve them, and the benefits, drawbacks, and best practices when involving them. The topic lists used can be found in Appendix A.

It proved difficult to find patients who had experienced a sentinel event and could participate in this study. At the time of the sentinel event evaluation, the patients were often no longer monitored by the healthcare organizations (dismissed, transferred, etc.) or deceased. Extra effort was made in a number of ways to recruit patients, for example via the Inspectorate, by contacting patient organizations, and by approaching experiential experts (former patients).

The interviews were conducted by the first four authors at a place preferred by the respondent(s) and lasted for 60–90 min. The interviews were recorded and transcribed verbatim. The transcripts made during the interviews were analyzed inductively by the authors (R.B., H.B., D.B., B.G.) following iterative grounded-theory techniques [25]. The results from the policy analysis were taken as the starting point of this process, *inter alia* because they formed the structure of the interviews. Codes were developed and evolved from this structure through repeated discussions between the authors. In particular, preliminary assumptions from the literature were reviewed with the data in an iterative process. This meant that we combined our inductive approach with deductive methods [26,27]. The definitive coding scheme can be found in Appendix B.

Three meetings where held with experts, including representatives from patient organizations and professional organizations in Dutch mental healthcare, to discuss our analysis at different phases of the research. These discussions were also used to interpret the results of the study and to validate the conclusions drawn by the researchers through member-check.

### 2.3. Medical Ethical Assessment

The study was conducted in accordance with the Declaration of Helsinki. All subjects gave their informed consent for inclusion before they participated in the study. The research protocol of this research was submitted for review to the Medical Ethics Committee (MEC, METC) of the Erasmus Medical Center (Rotterdam). The METC decided that the study did not fall under the Medical Research with Human Subjects Act (WMO) (MEC-2017-304). There was no objection to the execution of this study.

## 3. Results

### 3.1. Policy Documents

We now turn to our results, first of which are the outcomes of the analysis of the family policy documents, after which we discuss the sentinel event and suicide policy documents.

Table 2 provides an overview of our findings and shows how many policy documents explicitly included information on the three categories of involving patients and families: (1) informing; (2) hearing/listening; and (3) giving feedback. The results are described below for each type of policy document separately.

#### 3.1.1. Family Policy

In general, the family policy documents had more extensive descriptions of involvement than the sentinel event policy documents and the suicide policies. Family policies describe the active involvement of family members. This mainly concerns involvement in the treatment and care of a patient, rather than involvement in the evaluation of sentinel events. All policies explicitly stated that the involvement of family in all phases of the treatment process is of great importance. An example is:

“(Care organization) attaches great importance to the contribution of the client, legal representative, or relatives involved in establishing facts and describing events.”

(Family policy document of a mental healthcare organization)

In 10 family policy documents, it was emphasized that family is involved in the evaluation of the patient’s treatment. We have scored this as “giving feedback” (Category 3) because the aim is to request actual feedback from the family and to improve care. An example:

“(Care organization) endorses the fact that the bottlenecks and wishes expressed by family/close relatives can also improve care.”

(Family policy document of a mental healthcare organization)

In the family documents, attention is often paid to how to deal with privacy of the patient and how to deal with refusal by the patient to involve family. This is important because this is often perceived by professionals as a bottleneck for involving family [7,8].

#### 3.1.2. Sentinel Event Policy

Sentinel event policies seem to be focused on management; taking the correct procedural steps, and meeting the criteria of the inspectorate for reporting the event. Most sentinel event policy documents consisted of a list of definitions and a written or schematic step-by-step plan of the procedural and practical steps to be taken in the case of a sentinel event. The steps usually consist of who should be approached in which cases, who should be informed and within what timeframe, what should be reported and how (e.g., via the intranet), how to scale up within the organization, who is responsible for the investigation of the sentinel event and reporting on it, rules for the composition of the research team/committee, how follow-up of the sentinel event takes place, and who evaluates whether improvements have been implemented and how. The description of the role of patient and family during a sentinel event investigation, if mentioned, is limited to when the family needs to be informed in the process (Category 1).

Only one policy document mentions that the patient and family should be heard in an interview during the sentinel event investigation (Category 2).

In sentinel event policy documents from six organizations, nothing was mentioned about informing or contacting family or patients.

#### 3.1.3. Suicide Protocols

Within the suicide protocols, there is more attention to contacting family and relatives and aftercare. This is more about helping the family in their grieving process and answering all their questions (Category 1) and not so much on hearing their insights about what brought the patient to commit suicide or attempt to (category 2).

An example is:

“Most contacts with family logically take place in the first week, for example because employees attend the funeral or cremation of their client (in consultation with the family). It is important to schedule a conversation with family after six to eight weeks. In this interview, the results of the (first) internal suicide evaluation can be discussed with family and there is the possibility for relatives to ask questions about the treatment and counseling of the deceased client. The staff may also have questions for the next of kin that they would appreciate answers to.”

(Suicide protocol of a mental healthcare organization)

In summary, involvement of patients and families is described to a limited extent in all policy documents analyzed; which are often more focused on managing formal or internal processes of the care organization. Attention to involvement was paid mostly in the family policy documents. It focused primarily on involvement of family during general treatment of the patient. Less attention was paid in the sentinel analysis policy documents to the role of patients and families in the analysis of a sentinel event. In those policies, their role is mainly to be informed (Category 1) and less often to provide information or feedback (Category 2 and 3).

### 3.2. Interviews

Five overarching themes were identified in the coding of the interview transcripts (see Appendix B) and are discussed below using illustrative quotes from the interview data:

- involvement during treatment;
- definition of a sentinel event;
- limited involvement in practice;
- reasons in favor of involvement during sentinel event analyses; and
- reasons against involving patients and families in sentinel event analyses

Whereas the policy documents show the way in which mental healthcare organizations aim to define involvement (aftercare, providing information), such documentation does not tell us much about how involvement takes place in practice and does not show the tensions that are experienced in practice. Our interviews were meant to flesh out these tensions and to gain a better understanding of the practice of participation. We report on the main results from our interviews below, building on the five main themes we identified in our data. Where possible, we refer to the three categories of involvement as described above.

#### 3.2.1. Involvement during Treatment

The first theme we address is about the involvement of patients and family during treatment. When we asked our respondents about their experiences with involving patients and families in the analysis of sentinel events, most struggled to formulate a clear answer. Respondents, instead, focus on involving patients and their family in the daily process of care and treatment. A sentinel event and its analysis are seen as just the end of a long process that starts with the intake in a care organization. They feel that involving family and patients during daily care is more important.

Respondents have various reasons for the importance they attach to this involvement. We detail two here. Firstly, they say it helps to improve the quality of care in particular because involvement is said to help prevent sentinel events from occurring. For example, family members say they are able to pick up subtle signals about their relative who is not doing well:

“So she had compulsive thoughts. I noticed that straight away in her non-verbal communication. Actually in all sorts of small things. Then they upped her medication and valium. I said, ‘This isn’t going to help her enough. (...) I say, ‘you can keep her here, I’m not taking her home anymore, I’m no longer taking responsibility’. Then she got a psychosis on the spot.”

(Mother of a patient)

This quote also shows the attempts to convey these signals to professional carers. Such attempts, family members say, are often unsuccessful. Family members we interviewed often say that it depends on the professional they speak to if they are listened to or not. In cases where they are not listened to, they state that this resulted in a wide variety of dangerous situations. These include suicide and attempted suicide, as well as other types of dangerous behavior such as cycling on a freeway or committing an offense that puts other people at risk.

Involving patients and listening to them can also provide relevant information to help improve quality of care and prevent dangerous situations. One patient shared with us how the forced treatment she received in the healthcare organization led to her making an attempt to abscond:

“Yeah, and I’d be stamping and everything like that, and then they grabbed me: ‘Go to your room’. A man and a woman. The carers said, ‘And now to your room. If you don’t stop, you can’t leave your room for eight hours, or then you’ll get a jab or something,’ whatever. I thought, ‘Well, I’m pretty fed up with it.’ So I secretly got blankets, on my day off, I tied them all together. And the window wasn’t supposed to open, but it was one of the newfangled ones and I’d seen what to do. And when I’d opened it earlier, they’d seen that and pulled it closed. So anyway, I pulled that window open, blankets knotted together: well it worked. Normally I’m scared of heights, but not then—I just didn’t feel afraid. It was just tunnel vision. Then I went down, but those blankets came apart, so then I fell, on a platform luckily, with parking spaces beneath, a couple of stories high, not to the ground, (...) then I fell on my back.”

(Patient 1)

This patient felt unheard and frustrated, so she made an attempt to flee that resulted in a fall from a window with major physical consequences.

For patients and families to be taken seriously demands assertiveness in managing the tension between good care delivery and dependence of the patient on good care. Respondents point out that helping the family and patients to formulate their wishes can be important for this. That support can be provided by a family or patient counselor or members of a family or patient organization who can help families get their voices heard, which can be a fight and can depend on the person they are dealing with.

#### 3.2.2. Definition of a Sentinel Event

The second theme we identify in our data is that of defining whether or not a situation is a ‘sentinel event’. Different actors have different ideas on this which impacts the issue of involvement of patients and family in sentinel event analyses.

When something has gone wrong, various parties may have different opinions about whether a situation should be seen as a sentinel event. Family and patients may not share the opinion of the healthcare organization and the inspectorate on this issue. For example, according to family members we spoke to, a suicide or suicide attempt is always a sentinel event, whereas formally (following the legal definition) not all suicides or attempts are considered to be sentinel events. Differences in what is judged to be a sentinel event may occur in other situations too. Coercion measures can for example be experienced as sentinel events by patients. For care providers, coercion measures are often used to guarantee safety and therefore something that can prevent a sentinel event from happening. For example:

“...but he (the patient) doesn’t want that (coercion measures), so he’s less afraid of the sentinel event than the intervention to prevent it.”

(Patient counselor)

In case of such a conflict of opinion a formal response from the organization or the inspectorate that something is not a sentinel event according to the law can fall on deaf ears and cause irritation. One inspector said:

“So the jargon terms that we use, (...) certainly for people who are in mourning, are obviously incomprehensible. They often only trigger more anger and combativeness. Yes, that’s very difficult.”

(Inspector)

Definitional issues can arouse anger and combativeness in situations that are already prone to high emotions. Whether or not a situation is to be defined as a sentinel event depends on more than just what occurred in that situation. The director of one care organization also pointed out that when an event is quite controversial and it is expected to be published in the media, this can be a reason to treat the event formally as a sentinel event and report it to the inspectorate.

“But we also do it when there’s a lot of fuss being made.”

(Care institution director)

These examples demonstrate that defining an event as a sentinel event is not necessarily clear-cut. In practice the power to make such definitions generally lies with professionals, the care organization and the inspectorate, which leaves patient and family members with different opinions in a difficult situation.

#### 3.2.3. Limited Involvement in Sentinel Event Analysis in Practice

The third theme we identify is the limited involvement of patients and family in sentinel event analysis. In accordance with the results of our analysis of policy documents, family members have told us how their involvement in these analyses is very limited in both extent and depth. Professionals also tend to define the analysis as an internal process for the care organizations, leaving patients and families out.

If there is any involvement, respondents say that they generally look for input from family or patients at a late stage in the analysis, often after all other actors have already been heard. One director that we spoke to called the family briefly when she had made a list of her own findings about the situation, and the healthcare professionals involved with the event had already been interviewed:

“Then we draft the improvement measures, but at that point the family is no longer involved. At least everything (the event analysis) is done. No, the points for improvement are just outlines. If more points turn up, they’ll be in the report. And then worked out in detail. That’s without the family, but they can say what they think of the broader outlines.”

(Care institution director)

In this quote, it can clearly be seen how the family is left out at the moment details start to matter. While respondents do recognize that the perspective of family and patients can change the scope of analysis and the lessons learned, in several interviews respondents note that the perspective of patients and families not always adds up nicely with the perspective of other actors. They can also conflict. In such a case, our study shows that the contribution of family or patients quickly becomes devalued and questioned by healthcare providers or inspectors. When this happens patients and families are considered for example to be ‘overwhelmed by emotions’ (medical director).

Hence, involvement is only about ‘what (patients and family) think of it,’ not about their reflections on what might be improved in the future. As a result, the input of patients and family members is not used for formulating concrete points for improvement and neither do they get the chance to reflect on what went wrong. Throughout our study, we did not encounter any examples in which family or patients were involved on an equal footing with the professionals involved in evaluating what went wrong. In a similar vein, we found how narrowly choices are made about who is involved in the evaluation. Only one respondent talked about involving patients other than the patient directly involved in a sentinel event analysis (patients who witnessed the sentinel event). The results of the analysis are also only sporadically given to patients or family as feedback (corresponding to Category 3 of Table 2). One respondent from an organization indicated that they are thinking about sharing the findings of the analysis:

“So that’s what we do. But what we’ve recently discussed, the feedback for example on what has come from the analysis to the family, well, we don’t yet report that to them. And that’s the question, whether it might be useful to report it back to the family. Sometimes when the family really insist on it, but we don’t report that to them as a matter of course (...) And I think that’s the next step, that we also involve the family or patient in the evaluation and that we also give them the feedback, what came out of it (the evaluation), also to family and friends.”

(Director)

In conclusion, involving patients and families in sentinel event evaluation seems only to happen sporadically in practice, remaining limited to informing them (Category 1) and sometimes hearing what they have to say (Category 2) but at a late stage of the analysis. It is unclear whether their insights are really used for the analysis of the sentinel event. This limited involvement does not mean that respondents do not see the added value of involving patients and family, however involvement is also difficult in practice they feel. We now turn to these subjects in turn.

#### 3.2.4. Reasons in Favor of Involvement during Sentinel Event Analyses

If it is decided that a situation should be defined as a sentinel event, the question arises of whether patients and family members should be involved in its analysis. The fourth theme we identify labels the reasons our respondents provide in favor of such involvement. Below we sketch the three most salient reasons we came across.

Firstly, involvement in the form of aftercare is often mentioned as a reason for having contact with patients and families during the analysis of the sentinel event. In this view, it is important to support patients and families in dealing with what happened:

“It’s also a kind of duty for the care organization, to provide aftercare to the family. (...) To give them the opportunity to tell their story again, or to hear how everything happened. So that they can learn to cope with what has happened. Yes, in that sense it’s an extra reason for paying attention to the family and relatives.”

(Inspector)

In this reasoning, the involvement of patients and family members has added value in that it gives them a sense of recognition. Moreover, family members often want to know exactly what happened, especially in the case of a suicide. This knowledge could contribute positively to their grieving process. This corresponds to Category 1 of Table 2 (Informing them).

Secondly, patients and family members’ involvement is seen as valuable because they can provide information on top of the reports and conversations that have been conducted with the professional carers involved; this can improve the lessons drawn from the inquiry. One inspector expressed this as follows:

“(...) you need that family for the biography and the history. (...) The family is (therefore) indispensable for a proper analysis of the event, otherwise you’re only looking at the care provided and that’s the major problem, for the crisis services too. (…). Otherwise you’re taking snapshots and not seeing the movie.”

(Inspector)

This quote suggests that involving patients and families in sentinel event analysis is not only important for contextualizing the event but also for changing the scope of the analysis. Involvement, in this vain, concerns the run-up to the sentinel event and the context in which it took place, instead of focusing only on what exactly happened at the time of the event. Involving family is therefore seen as helping to provide the analysis with other questions and thus other conclusions and lessons from the analysis:

“… Because you’ve seen a few reports on sentinel events and you know you’ll get answers based on the questions you put in. If you make your analysis narrow enough, nothing seems to have gone wrong.”

(Family counselor)

These examples refer to Category 2 of Table 2 (Hearing patients and families about their experiences of the event).

Third, involvement of patients and families in the analysis is seen as valuable for being able to prevent legal proceedings or escalation of the situation in other ways. Respondents say that if no contact is made with involved patients and families after a sentinel event, this may evoke the feeling that the organization is trying to hide something. In that case, some of them continue to search for ways to make themselves heard, for instance by contacting the inspectorate, filing complaints, or starting disciplinary procedures. Being open about sentinel events is thus seen as valuable in preventing escalation to formal procedures:

“It’s better to share the real story with each other than to follow formal pathways. That’s pretty much our approach.”

(Family counselor)

Engaging in dialog with each other is thus felt to be beneficial for both patients and family and the care organization. However, reasons against involvement are also given.

#### 3.2.5. Reasons against Involving Patients and Families in Sentinel Event Analyses

The fifth and final theme involves the reasons our respondents provide against the involvement of patients and family members. From our interviews we can identify a number of challenges that seem to hamper involvement, we discuss the five most salient challenges here.

Firstly, respondents refer to the complexity of sentinel events in mental healthcare. This complexity lies in part in the nature of the sentinel events in this sector where—unlike in somatic care—patients often play a role in instigating the sentinel event (e.g., violent behavior, suicide attempts).

Secondly, family or patients do not always want to be involved in the analysis of a sentinel event. In cases of suicide, the patient is no longer there and the impact on the family is potentially enormous. In such cases, the family sometimes do not want anything to do with the organization, which of course makes involvement in the analysis impossible:

“You often see it at the actual funeral. Because if someone was admitted here, everyone will have been very involved with the patient. So if they commit suicide, we always ask whether we should go to the funeral or not. And some families say that they are at any rate glad that they were helped and it’s a shame it went wrong, but you’re welcome to come. But of course there are families who do say, ‘this should never have happened. We never want to see you again’. So (...) how families react can be very different.”

(Psychiatrist)

This major impact means that family and patients are not always or immediately able to participate in a formal analysis of a sentinel event. Even after a few months, family and patients may still want to avoid the situation and contact with the care organization. Perspectives on time for grieving and recuperation differ from the formal deadlines of the analysis process, which are often seen as coming too soon. For patients this might even be a more important bottleneck for being involved. One patient told us that it took a long time for her to be able to talk about what happened:

“I just think later, after waiting a few years first until it all… It really takes a few years before the psychosis is really gone; it‘s not right away, but I think a few years later, in my case five years later, that it’s good to think about it again.”

(Patient)

To be able to continue and move on, it might not be in the patient’s or family’s best interest to participate in a formal analysis. The family members, patient counselors, and family counselors we interviewed underline that participation should therefore not become an obligation for patients and families. They argue that a sentinel event evaluation does not have to be a priority for the patient and family, and this needs to be given its legitimate space. In addition, the family could also be absent, no contact can be made with them, or the contact between family and patient may have been lost years ago.

Thirdly, in addition to family members and patients who are not willing or at a certain point in time not able to participate, respondents from care organizations also note they feel patients and families lack the necessary knowledge to be able to contribute. In several interviews, respondents noted that family members and patients, certainly in the context of the more technical root cause analysis of the causes of a sentinel event, do not have sufficient expertise to participate. Although organizations generally use an open format in the analysis, the conclusion is sometimes that the evaluation method hampers involvement.

“(...) PRISMA (Prevention and Recovery Information System for Monitoring and Analysis, a method of root cause analysis) is an instrument mainly for seeing if mistakes were made in the organization. So very often you have patient-related factors as a base cause an, well, whether they were manic or angry doesn’t really matter. So from the patient’s perspective, a PRISMA sounds a bit brutal and is irrelevant.”

(Psychiatrist)

The question underlying this discussion is who assesses whether involvement of patients and families is non-functional or undesirable in a sentinel event analysis and how that decision is made.

Fourthly, the privacy of the patient and professionals might hamper family involvement. Because of this, organizations do not always share information about the sentinel event with the family. Sometimes the patient explicitly stated that they did not give permission for information to be shared with family. In other cases, it was assumed that a patient has not given permission to share information with family. Nonetheless, the use of privacy as an argument not to involve family is an important source of misunderstanding and frustration among the family members we spoke to.

Fifthly, it can be difficult for providers to involve patients and families because this requires them to be open about what happened. This can be perceived as difficult because of the large impact of a sentinel event on the professionals involved:

R1: “Yeah, admitting everything that went wrong. And being able to investigate how and why properly.”

R2: “Yes, that’s very difficult, isn’t it? How can you be transparent if there‘s a family and you’re discussing it afterwards? And that someone wants to be open and wants to search, you do not. That’s way too fraught. Especially in events like these.”

(Medical director and psychiatrist)

Because of the sensitivity of the events, some respondents felt that being open and protecting the privacy of healthcare professionals hampers involvement. Being open is related to a sense of insecurity, being afraid of the reaction of the patient, negative media coverage, and the possibility of a disciplinary complaint being filed. Such considerations turn sentinel events into an internal matter of the care organization. For care providers too, it is a serious matter and a safe culture is said to be needed for proper reflection. Care professionals we interviewed link the lack of transparency to the need to create a safe and blame-free culture for professionals to share and report their mistakes, experiences and emotions. This is also seen as essential for encouraging learning from mistakes. Respondents from care organizations feel the need to protect the professionals, as no formal attention is given to them:

“So the inspectorate only wants to know if the patient is the victim. Whereas in ninety-nine out of a hundred cases, the staff member is the victim.”

(Medical director)

This example also shows that sentinel events can have a major impact on care professionals too, which also makes it more difficult for them to involve patients and their family and to be confronted by them.

## 4. Discussion

To our knowledge, this is the first study that explores the experiences of involving patients and families in the analyses of sentinel events (including suicide and attempted suicide) in mental healthcare. Our results show that the involvement of family and patients can be improved in practice, both during treatment and during sentinel event analyses; the importance of the first was emphasized by respondents we talked to. However, as of yet there appears to be little knowledge and experience in practice about how to deal with patient and family involvement after a sentinel event, especially when focusing on mental healthcare.

The policy documents of care organizations do not say much about this issue. The description of the role of patient and family during a sentinel event analysis is limited to when the relatives should be informed in the process, the other categories (hearing/listening and giving feedback) are generally lacking. In six out of the thirteen sentinel event policy documents analyzed, nothing was found about how to involve patients and family members in a sentinel event analysis. It is important to note, for these results, that the non-responding care organizations may have developed fewer policies and that involvement of patients and families in the policy documents may be even more limited. However, this lack of attention in the policy documents does not mean that the involvement of patients and families in sentinel event analyses is not endorsed by our respondents, including those representing care organizations. They stress the importance of aftercare, state that involvement can help the grieving process of patients and families and patients and family can provide essential information for the analysis which can be used for improvement, and can prevent escalation to other formal processes after a sentinel event.

One of the main themes in our interview data focuses on the notion that involvement during the analysis of sentinel events is inseparable from involvement during assessment and treatment. Respondents generally view the sentinel event analysis as ‘only’ the end stage or potential end stage of a long process of co-producing care that starts with the intake of a patient. Involvement of patients and family members from the start of treatment can even prevent sentinel events, according to our respondents. Recently developed national guidelines stress the importance of early involvement of family in the treatment process, and give concrete suggestions about when and how family should be involved [10,20,28]. Our respondents recognized a shift towards more involvement of others during treatment, but also that this is just beginning to be implemented in the Netherlands.

When we turn to involvement after sentinel events our results show a number of complexities. This begins with the definition of a sentinel event. Respondents have different perspectives on what constitutes a sentinel event. Families almost always consider suicide (attempts) to be a sentinel event, even when it is not related to the quality of care provided (which means that formally organizations and the inspectorate do not consider it a sentinel event). Moreover, patients may perceive coercion measures (which are aimed to prevent formal sentinel events) to be sentinel events in itself. This definition issue is strongly present in the context of mental healthcare and closely linked to issues of power and coercion. Other research also shows that the definition of a sentinel event can conflict between different stakeholders.

Our study shows that sentinel events in mental healthcare are more complicated for other reasons as well, which further complicates the involvement of patients and family after such events. Compared to hospital care for example, sentinel events in mental healthcare are more often related to the behavior of the patient, for example violent incidents or suicide [29]. In the case of events involving violence and aggression, for example, professionals are more often the direct victim according to our respondents. We find indications that the active role of the patient in a sentinel event in mental healthcare makes the analyses different to cases where the event has a more straightforward technical cause [30].

The above suggests that one standard definition for all sentinel events in all healthcare sectors may not be not appropriate. As other researchers also argue, patients’ definitions of harm and incidents should also be included in patient safety analyses, even when they concern non-clinical issues or when no damage occurred [31,32]. This also suggests that sentinel event analyses alone may not be the best method for learning about such behavioral incidents, but that other methods are needed for instance looking at how to recognize the warning symptoms that often precede events with a behavioral component [33,34]. Our results show that this is an important issue where the insights of patients and family can contribute to improving care. For example by providing ‘soft intelligence’, information about blind spots, that care providers are unaware of [35].

However, in this study, we find little evidence of such contributions. Respondents from care organizations told us that involving patients and families during a sentinel event analysis is complicated for a variety of other reasons then the definitional issues concerning sentinel events. Amongst these are the difficulties experienced by care professionals to be open about what has gone wrong, the fear of legal consequences for staff, and the desire to protect staff. In addition our results show that not everyone can participate, or wants to be involved (directly) after a sentinel event. In addition, professionals highly value the autonomy and privacy of patients, which can also exclude family members [7].

Our study shows that there are questions and problems to solve for which no easy answers and solutions are available. One such issue is that the perspectives of patients and families can conflict with those of healthcare organizations and the inspectorate. This means that when the decision is made to involve patients and families, reflection is needed on how to deal with possible conflicts of perspectives [36]. Involving patients and families in sentinel event analyses appears to be challenging, but also to have important benefits. Some lessons learned in other care sectors may help overcome some of the challenges. Recent studies, in hospital care, have focused on openness towards patients and their families by professionals after incidents and sentinel events. Important themes are the recognition of the error, apology, professional support, timeliness, and clarity of communication [32,37,38,39,40,41,42]. This could prevent formal complaints and claims from being lodged. This also fits with the shift in culture in healthcare from a ‘blame culture’ towards a ‘just culture’ in which employees should be able to share important safety information without fear of repercussions and they know where the boundary is between acceptable and unacceptable behavior [4]. In addition, research in hospital care suggests identifying the needs of patients and family in advance of a sentinel event analysis, giving them a clear role, involving them as early as possible in the analysis and also giving them more time if they need it, and having the process supervised by trained facilitators can help involvement [1,2]. Respondents also pointed out that family and patient counselors can play a role in supporting them in the process of sentinel events. In addition, there are ideas for organizing participation in sentinel event analyses by other means, for example through training programs for doctors, through special committees, or patient representatives [1,2]. These ideas require further research, especially in the mental healthcare setting.

These insights might provide future directions for the involvement of patients and families in sentinel events in mental healthcare. This explorative study brought a first picture about this subject. It could be further studied by evaluating ongoing cases of sentinel event analyses, in order to gain greater understanding about these processes and assessing the experiences, feelings, and opinions of involved persons directly during the analyses.

## 5. Limitations

There may be a risk of response bias in the proportion of healthcare organizations that did not respond to our question about policy documents. Furthermore, recall bias may play a role, as sentinel events may be uncommon at the level of mental healthcare organizations, and respondents may only have experienced such an event some time ago. As described in the methods section, patients who experienced a sentinel event were difficult to reach. We therefore approached various patient representatives, counselors, and family members who gave a picture of the patients’ perspective. We aimed to include all types of events in order to gain a rich and deep understanding of the experiences of involved persons. We did not make a distinction in the type of sentinel event or the severity of the events, which of course may relate to the types of reactions of patients and family. Future studies could take this into account. It should be noted that although our qualitative approach fits well with the explorative nature of the study and enabled the collection of rich and detailed data, that are in line with the existing body of research, caution is advised when translating the results to the wider mental healthcare sector or other care sectors.

## 6. Conclusions

In summary, we obtained notable insights from this study: stakeholders in mental healthcare find it important and valuable that patients and families are involved in the analyses of sentinel events. However, little attention is paid to such involvement in the policies of care organizations, and instead respondents link it to involvement in routine care. Furthermore, in practice, involving patients and/or families after a sentinel event in mental healthcare seems only to happen to a limited extent. Aftercare and information provision are the most common forms of involvement, and care organizations make sentinel event analysis an internal process, leaving little room for patient and family perspectives. The sometimes different perceptions and conflicting perspectives of patients and families compared to the perspectives of the care organization raise questions about the role of patients and families in the sentinel event analysis. The analysis of what has happened; including the run-up to the event as described by patients and families, can also lead to different conclusions, even about whether or not the event should be formally classified as a sentinel event. However, if something goes wrong in the experience of the patients that does not fall within the legal definition, this can still be discussed within the organization and learned from.

In other care sectors, greater experience has already been gained with involving patients and families in sentinel event analysis. Some initiatives are introduced with positive outcomes, such as involving trained facilitators or patient representatives in the sentinel event analysis process which may provide some guidance for the mental healthcare sector. There is no one-size-fits-all answer to how patients and families can be involved better in these sentinel event analyses. Nevertheless, it seems beneficial to focus on involving them at the start of treatment and throughout the whole treatment.

## Figures and Tables

**Table 1:** Score list for the policy documents.

Year that policy was drawn up.
Year that policy was updated.
Frequency of review/evaluation of policy.
Sentinel event policy: the patient/family are informed.
Sentinel event policy: the experiences of the patient/family are listened to.
Sentinel event policy: the patient/family are given the opportunity to give feedback.
Family policy: the patient/family are informed.
Family policy: the experiences of the patient/family are listened to.
Family policy: family are given the opportunity to give feedback.
Suicide protocol (if included): the patient/family are informed.
Suicide protocol (if included): the experiences of the patient/family are listened to.
Suicide protocol (if included): the patient/family are given the opportunity to give feedback.

The results are presented per type of policy document. Counts are shown for the occurrence of a specific category in the texts of the policy documents.

**Table 2:** Scores of the extent to which involvement of patients and families is described in the policy documents of mental healthcare institutions.

Category of Involving Patients and Families	Extent of Involvement in Family Policy (*n* = 12)	Extent of Involvement in Sentinel Event Policy (*n* = 13)	Extent of Involvement in Suicide Policy (*n* = 5)
**1.** **Informing**	12	7	4
**2.** **Hearing/listening**	11	2	3
**3.** **Giving feedback**	10	0	2

## Appendix A

Topic lists for patients/families and professionals

Topic list for patients/family

Introduction: Explanation of research (especially: role of inspectorate, funder but independent), explanation of interview (audio recording, confidentiality, etc.)

Who is who: Where have you or your relative been admitted? And for what?

Sentinel event: can you explain what happened? How have you been informed about this? By whom and when? Would you like to see this differently?

Involvement in sentinel event analysis: Have you been involved in the analysis? If so, at what time? How did this work? How did you experience this? How could this have been better?

Involvement in incidents: Broader than a formal sentinel event: what role do relatives/patients get ‘if things go wrong’? Why is that important? What is changing?

Concretization: (if it does not happen by itself) What are your experiences with involving patients/relatives in the most recent sentinel event? What went well, what could be better? Other experiences that you want to share?

Interaction with Inspectorate: How is the contact with the Inspectorate, institution and family after a sentinel event report? Have you ever had contact with Inspectorate?

Involvement in treatment relationship: How important is the role of family members/patients in the treatment relationship?

Final questions: have we not discussed something important? Documents? Subsequent questions/contact?

Topic list for professionals

Introduction: Explanation research, explanation interview (audio recording, confidentiality, etc.)

Definition and meaning of the sentinel event: How/when is something considered to be a sentinel event? How often does this occur? What does that mean for you and your employees? How does this work?

Involvement in sentinel event analysis: How do relatives/family /patients formally perceive a sentinel event? How/are they involved in the analysis (follow-up/formal investigation/...)? How does that work in practice? (call/conversation/written ....) What are the considerations? Is this drawn up in policy and what exactly does that policy prescribe? What are the experiences with this involvement? What goes well? Which tensions are occurring?

Involvement in sentinel events: what role do family/relatives/patients ‘when things go wrong’? Why is that important? What is changing?

Concretization: (if it does not happen by itself) What are your experiences with involving patients/relatives in the most recent sentinel event? What went well, what could be better? Other experiences that you want to share? Are there other initiatives in the area of involvement of family members and patients?

Interaction with Inspectorate: How is the contact with the Inspectorate after you make a sentinel event report? How is the involvement of patients and relatives in sentinel events communicated with the Inspectorate? Does it ever happen that the Inspectorate criticizes the way in which family members are involved? Should the Inspectorate encourage this, why and how?

Role of other actors: e.g., professional/informal carers, police, municipality, media? Influence on whether or not, and how, of involving family and patients?

Involvement in treatment relationship: How does the role of family members/patients in the treatment relationship usually take shape? Who determines that? (#check formal requirements). What is important in this?

Case selection: ‘Best practices’ explanation idea behind the next step, possibly known cases that we could look at in more detail? Tips/tricks how to approach patients and relatives?

Completion: have we not discussed something important? Documents? Subsequent questions/contact?

## Appendix B

Codes used in the analysis of the interviews

Nature of method of sentinel event analysis (PRISMA)

Involving other patients

Involvement as a policy

Involvement as aftercare

Involvement as feedback of information.

Involvement in care—sentinel event analysis

Involvement in care—daily care

Involvement in care—preventing calamities

Involvement is normal

Involvement is a lot of work

Involvement through others (family/patient counselors)

Involvement at policy level

Definition of sentinel event

Emotions of personnel

There is no family/relative

Experiential experts

Family determined (level of) involvement

Family does not want contact

Family: losing contact

Family: double role (support/problem)

Culture in mental healthcare institution

Media sensitivity

Patient does not want family to be involved

Patient does not want personal involvement

Personnel as victim

Reasons not to involve—‘difficult’ for assistance

Reasons not to involve—‘too sensitive subjects’

Reasons not to involve—lack of knowledge

Reasons not to involve—legal conseq.

Reasons not to involve—not relevant

Reasons not to involve—practical problems

Reasons not to involve—privacy of the patient

Reasons not to involve—privacy of personnel

Reasons not to involve—too emotional

Reasons not to involve—too much burdening.

Reasons not to involve—wrong questions

Reasons for involvement—give recommendations

Reasons for involvement—informing

Reasons for involvement—reflection (beyond)

Reasons for involvement—connecting

Reasons for involvement research—additional source of information

Reasons for involvement research—avoid escalation

Reasons for involvement research—processing

Role of Inspectorate

Type of sentinel event

Tensions—conflicting perspectives

Tensions—complex matter

Tensions—complex care

Tensions—procedures/emotions

Tensions—structural developments mental healthcare

Specificity of mental healthcare
